# A Report on the Origin and Therapeutic Properties of Cundurango

**Published:** 1873-08

**Authors:** 


*A Report on the Origin and Therapeutic Properties of Cundurango.* By W. S. W. Ruschenberger, M. D., Medical Director U. S. N., President of the Academy of Natural Sciences Philadelphia, etc. Washington: Government Printing Office, 1873. Published by Order of the Navy Dep't.

This report is one which was presented in April 1872, to the Surgeon-General of the Navy on the Origin and Therapeutic Properties of Cundurango. The report on the origin of the drug is based upon that of Dr. Ayers, Assistant-Surgeon U. S. N., who was directed by the Surgeon-General to collect botanical specimens and such information as he could obtain relating to Cundurango.

Dr. Ayers was at the time stationed on the coast of Peru and at once proceeded to the locality where Cundurango was to be found. After traveling over a large portion of country, being absent from his ship some thirty-nine days, Dr. Ayers was able to forward to the Bureau of Medicine and Surgery Cundurango, leaves, flowers and fruit, comprising ten varieties.

The origin of the reputed cure of Cancer by Cundurango seems to be shrouded in several conflicting reports. The reports of cases of cure by South-American Physicians shows that they differ somewhat from the generally accepted views of cancer. Dr. Eguiguren, who seems to have first tested its value in the year 1869, remarks, that the cases which he treated, "were already in the form of fungus harmatodes and none in the state of rawness."

The story is told of the Indian woman who wishing to give her husband eternal relief from an "internal cancer" whose existence we are left to infer she recognized herself, administered a decoction of the wood of cundurango, in stead of the fruit, which was said to be an active poison. Imagine her surprise to find that her husband at once began to rally. Another version of the story places the affection external rather than internal and characterizes it as being "sores which emitted an intolerable odor and were covered with maggots." In both cases there was marked improvement. The Archbishop Rio-frio informed Dr. Ayers that the man had leprosy. There is also a tradition among the people of his native place that it was syphilis of a severe character of which he was cured. Be this as it may, he had been dead over forty years when Dr. Ayers was there, so the discovery of the virtues of cundurango was not so *recent* after all.

The report cites cases, reported by several South American Physicians, of cancer, syphilis, åsthma, etc., in which cundurango was used with reputed benefit.

The most interesting part of the report is that concerning the plant itself. According to Dr. Destruge, Cundurango belongs to the order Asclepiadaceæ, third tribe, which corresponds to Asclepiadaceæ Vera, first division, Astephanæ, whose characters are that the limb of the corolla is without scales, and the stamens without appendage or corona.

This division comprehends only five genra in none of which can cundurango be classed, it therefore forms a new genus. As Dr. Destruge has not given a name to this genus it is suggested by the author that it be called Pseusmagennetus equatoriensis (from ψευσμα, a lie, a fraud, and γεννητης, a parent, a producer,) which seems to us a very appropriate name.

The specimens forwarded by Dr. Ayers were labeled:—Cundurango blanco, two varieties of cundurango de paloma, Cundurango de tumbo grande, de platano, de tumbo chico, cascarilla, saragosa, amarillo, and a variety growing near Guayaquil, ten varieties in all which are described to some extent in the text. Cundurango blanco or white cundurango is the variety which is used as a cancer cure.

An analysis of the plant by Dr. Antisell published in the *Medical Record* shows that the therapeutic position of the plant is among the aromatic bitters.

It seems rather strange that after being known in South America for over forty years it should be suddenly introduced into the U. S. as a new remedy for cancer, upon the testimony of a few ignorant Indians.

We have been censured by the press for speaking too strongly against this remedy it being insisted upon that it was capable of curing cancer. We recommend that those who were inclined to blame us read this report, in which they will find that upon careful examination of the cases in S. America, Dr. Ayers was of the opinion that the diagnosis was not very *carefully* made in the cases I reported *cancer*, they will also learn that upon trial both in this country and England by careful and observing physicians the opinion was arrived at that as a cure for cancer cundurango was perfectly inert.

The report is embelished by twenty-one full page photographs of cundurango leaves, wood, flowers and fruit, and by one of the leaves of a plant called Chininiga obtained by Dr. Ayers in S. America which is administered there as a remedy for syphilitic skin diseases. As a scientific work its only value lies in the description of a new plant and in the fact that it will remain as a monument of the credulity evinced by the American people in accepting cundurango on so small and unreliable a recommendation as a remedy for Cancer.

