# Errata

**Published:** 1873-09

**Authors:** 


ERRATA.—In Vol. XIII, No. I, Aug., 1873, page 36 last line for "harmatodes," read "hæmatodes." Page 37, last line for "cases I reported *cancer* etc, read "of reported *cancer*."

